# High Granulocyte-Macrophage Colony Stimulating Factor to Interleukin 10 Ratio and Marked Antioxidant Enzyme Activities Predominate in Symptomatic Cattle Naturally Infected with *Candidatus Mycoplasma haemobos*, *Theileria orientalis*, *Theileria sinensis* and *Trypanosoma evansi*

**DOI:** 10.3390/ani11082235

**Published:** 2021-07-29

**Authors:** Onyinyechukwu Ada Agina, Kim Tho Cheah, Nurul Syahirah Ahmad Sayuti, Mohd Rosly Shaari, Nur Mahiza Md Isa, Mokrish Ajat, Mohd Zamri-Saad, Mazlina Mazlan, Hazilawati Hamzah

**Affiliations:** 1 Department of Veterinary Pathology and Microbiology, Faculty of Veterinary Medicine, Universiti Putra Malaysia, Serdang 43400, Malaysia; cheahkimtho@gmail.com (K.T.C.); nurulsyahirah0806@gmail.com (N.S.A.S.); nurmahiza@upm.edu.my (N.M.M.I.); m_mazlina@upm.edu.my (M.M.); 2 Department of Veterinary Pathology and Microbiology, Faculty of Veterinary Medicine, University of Nigeria, Nsukka 410001, Nigeria; 3 Animal Science Research Centre, Malaysian Agricultural Research and Development Institute, Serdang 43400, Malaysia; rosly@mardi.gov.my; 4 Department of Pre-Clinical Sciences, Faculty of Veterinary Medicine, Universiti Putra Malaysia, Serdang 43400, Malaysia; mokrish@upm.edu.my; 5 Centre for Ruminant Diseases, Faculty of Veterinary Medicine, Universiti Putra Malaysia, Serdang 43400, Malaysia; mzamri@upm.edu.my

**Keywords:** cytokines, serum, ELISA, asymptomatic and symptomatic cattle, blood parasites, oxidant/antioxidant

## Abstract

**Simple Summary:**

The aim of this study was to evaluate the proinflammatory (interleukin 12, GM-CSF, interferon-γ) to anti-inflammatory (interleukins 10, 4) cytokine ratios, oxidant level (malondialdehyde) and antioxidant enzyme (superoxide dismutase, glutathione peroxidase) activities in sera from symptomatic and asymptomatic cattle infected with blood parasites relative to clinically healthy cattle. Elevations in serum GM-CSF level was recorded in both symptomatic and asymptomatic cattle infected with blood parasites. Cattle naturally infected with *C. M. haemobos* and *T. orientalis* but without clinical signs had anti-inflammatory cytokine immune responses characterized by low proinflammatory to anti-inflammatory cytokine ratios which favor parasite persistence and resistance in the host. Serum activities of antioxidant enzymes (superoxide dismutase and glutathione peroxidase) and malondialdehyde concentration were elevated in all the symptomatic and asymptomatic blood parasite infected cattle groups. Therefore, a high GM-CSF:IL10 response alongside marked antioxidative enzyme responses were predominant findings in cattle with clinical signs of natural blood parasite infections.

**Abstract:**

The aim of this study was to measure the serum proinflammatory (IL-12, GM-CSF & IFN-γ) to anti-inflammatory (IL-10, IL-4) cytokine ratio, oxidant (MDA) level and antioxidant enzyme (SOD; GPx) activities after blood parasite infections. The blood and serum samples were obtained from 130 cattle and screened for identity of the infecting blood parasites by conventional PCR. The following blood parasite species were detected: *Candidatus Mycoplasma haemobos* (70/130); *Theileria orientalis* (65/130); *Theileria sinensis* (32/130); *Anaplasma marginale* (49/130); *Anaplasma platys* (7/130); and *Trypanosoma evansi* (4/130). The GM-CSF/IL-10 ratio showed significantly higher values in all the symptomatic blood parasite infected cattle groups except for symptomatic *A. platys* infected cattle groups. Anti-inflammatory cytokine immune responses were notable findings in symptomatic and asymptomatic cattle infected with *C. M. haemobos* and *T. orientalis* characterized by low serum IL-12:IL-10, IFN-γ:IL-10, IL-12:IL-4 and IFN-γ:IL-4 (*p* < 0.05). Therefore, high serum GM-CSF:IL:10 in the symptomatic blood parasite infected cattle, low serum IL-12:IL-10, IFN-γ:IL-10, IL-12:IL-4 and IFN-γ:IL-4 ratios in asymptomatic cattle, high MDA level, and increased antioxidant enzyme activities could be useful predictive tools for outcome of natural blood parasite infections in cattle.

## 1. Introduction

Vector-borne diseases such as those caused by *Anaplasma*, *Theileria*, *Trypanosoma*, haemotropic Mycoplasma (hemoplasma) species are common in tropical and sub-tropical parts of the world. This is ascribed to the constant interaction between these haematophagous vectors and host, and a favourable hot and humid climate for breeding and survival [1,2]. The blood parasites invade or attach onto the erythrocytes thereby inducing haemolytic anaemias which may be mild, moderate or severe depending on the number of infecting pathogens, host immunity, nutrition, age and breed [3]. Invasion or attachment of the parasites onto blood cells usually occurs following evasion of the immune system by these parasites, through their respective evading mechanisms [4]. Recovered animals may become asymptomatic carriers and become sources of infection for naïve or immunocompromised cattle recently introduced into a herd. Clinical signs associated with blood parasite diseases include mild to severe anaemia, fever, weight loss, increased ocular discharge, decreased milk production in dairy herd, neurological signs such as convulsion and tremors, anorexia, depression, abortion in severe cases, coagulation disorders, jaundice, haemoglobinuria, oedema and reproductive abnormalities. Route of infection is mainly by the bite of ixodid ticks (*Haemaphysalis, Boophilus, Rhipicephalus, Amblyomma*) or biting flies (*Stomoxys calcitrans*) in the case of *Trypanosoma evansi.* Transplacental and/or colostral transmission have also been documented [5].

The host immune response (innate or acquired) to invading pathogens usually results in inflammatory processes that are mediated by cytokines [6]. These immune responses (cellular and humoral) are also primarily based on the transmission of cytokine inhibitory or stimulatory signals [7]. Cytokines are genuine pleiotropic molecules that play a role in immune response via autocrine and paracrine signaling in order to regulate and physiologically influence specific target cells. They are synthesized or expressed by different cell types such as monocytes, macrophages, T lymphocytes (T Helper 1 and 2), monocytes, endothelial cells, fibroblasts, astrocytes and microglia cells following induction by pathogens [8]. These cytokines, also known as protein hormones, act as messengers between the local site of injury and the liver cells, which produce the acute phase proteins, leading to induction of acute phase response in early inflammatory processes (Reviewed in [9]). Inflammatory processes usually involve increased vascular permeability and blood flow which is accompanied by leukocytic infiltration (recruitment of inflammatory cells) and secretion of pro- and anti-inflammatory cytokines. Sometimes, inflammation may go unnoticed or its not properly displayed with obvious clinical signs resulting in subclinical infection which can affect performance in food animals [9,10]. Therefore, in order to establish a potent immune response, there must be a dynamic balance between the pro- and anti-inflammatory cytokine responses that allow for restoration and maintenance of immune homeostasis, by acting as chemical mediators to bridge the innate and adaptive immunity in inflammatory conditions associated with infections [11]. Increase in pro-inflammatory cytokine levels such as interleukin-12 aids in blood parasite clearance from peripheral blood, while IL-10 plays a central role by suppressing the inflammatory process. It inhibits cytokine synthesis by T helper 1 cells and inhibits the cytotoxic activities of monocytes and macrophages as well as synthesis of proinflammatory cytokines and acute phase proteins and thus, has been regarded as the major cytokine that suppress or regulate the inflammatory process and immune response [12]. The aforementioned properties of IL-10, therefore, encourages blood parasite persistence in the host, and consequent carrier state in animals. Cytokines such as interleukin-12, IFN-γ, TNF-α are produced during the acute stages of infection. Due to increase in different types of cytokines at different stages of infections, cytokines are proposed as potential biomarkers of infection. They also contribute to the pathology observed in certain diseases. A shift from the production of proinflammatory cytokines at the beginning of infection to anti-inflammatory cytokines in late or chronic infection is correlated with the ability of the host immune system to reduce parasitaemia and pathology, respectively [13]. Granulocyte-macrophage colony stimulating factor (GM-CSF), also known as colony stimulating factor 2 (CSF-2), is a monomeric glycoprotein and a product of cells of the innate immune system (macrophages, natural killer cells, mast cells, T cells, endothelial cells and fibroblasts), T cells, activated during the inflammatory or pathologic conditions upon receiving immune stimuli, and a cytokine with immunomodulatory activity ([14]; reviewed in [15]). It upregulates the adhesion molecules on fibroblast and endothelial cells. As a white blood cell growth factor, it is involved in the regulation of myelopoiesis by promoting myeloid cell development, maturation and survival [16]. It stimulates differentiated haematopoietic precursor cells into monocytes and granulocytes (neutrophils, eosinophil and basophil) [17], and plays an active role in the proliferation and differentiation of monocytes into macrophages and dendritic cells [18]. Amongst IL-4 and IL-13, GM-CSF is the major cytokine that stimulates the in vitro production of dendritic cells [18]. It regulates T cell, dendritic cell functions and antigen presenting cells activation [19]. GM-CSF has an effect on mature cells of the innate immune system. For instance, GM-CSF enhances neutrophil migration into inflammatory sites and alters the receptors expressed on their surface [20]. Therefore, it is part of the immune/inflammatory cascade, by which activation of a few macrophages can rapidly lead to an increase in their numbers, a mechanism that is important for fighting infection and enhancing their functions in host defense. Macrophages and granulocytes are stimulated by GM-CSF to secrete plasminogen and produce various pro-inflammatory cytokines such as TNF-α, IL-1 and IFN-γ, thereby enhancing their microbicidal and phagocytic activities [18]. Goldstein et al. reported its use as therapy for treatment of innate immunity disorders such as inflammatory bowel diseases. It is also approved for treating bone marrow transplant graft delay or neutropenia in patients after induction of chemotherapy in cancer patients (reviewed in [21]). Its use as an adjuvant in vaccine stimulates cellular and humoral immune response with remarkable T and B cell proliferation and differentiation. The imbalance between proinflammatory and anti-inflammatory may contribute to the development of certain pathologies such as development of multiple organ dysfunction in humans [22].

Reactive oxygen species (ROS) and free radicals such as superoxide radical (O_2_^−^), hydrogen peroxide (H_2_O_2_) and hydroxyl radical, are constantly generated during metabolic processes that occur in cells/tissues. Under physiological processes, there is a balance in the cellular ROS generation and activity of antioxidant enzymes and non-enzymes, and other redox molecules. However, a disturbance in the oxidant/antioxidant equilibrium, seen in blood parasite infections (e.g., theileriosis) can initiate lipid peroxidation which in turn leads to free radical chain reaction that causes cell injury and death [23]. Antioxidant enzymes such as superoxide dismutase (SOD) and glutathione peroxidase (GPx) are the first line of defense against reactive oxygen species mediated injury [24,25]. Superoxide dismutase catalyzes the dismutation of superoxide anion free radical into molecular oxygen and hydrogen peroxide and depletes the level of superoxide radical (O_2_^−^), whose increased concentration can cause excessive damage to the cells. Several studies have revealed the therapeutic properties of SOD, and its role as an anti-inflammatory agent and treatment of cancer (reviewed in [26]. Glutathione peroxidase detoxifies endogenous and exogenous reactive oxygen species and eliminates xenobiotics in cells. In addition, its function is to prevent oxidative damage of cellular constituents, thus its deficiency promotes oxidation of protein and deoxyribonucleic acid (DNA) (reviewed in [25]). Malondialdehyde concentration (MDA) is used as a marker of lipid peroxidation, an indicator of oxidative stress and it is derived from polyunsaturated fatty acids oxygenation [27]. To date, only very few available literatures on the alterations in cytokine profile and oxidant/antioxidant levels following natural blood parasite infection in cattle [6,23,24]. Therefore, this present study compares the serum concentrations of proinflammatory (IL-12, GM-CSF and IFN-γ) and anti-inflammatory (IL-10, IL-4) cytokines, proinflammatory to anti-inflammatory cytokine ratios, oxidant (malondialdehyde) and antioxidant enzymes (superoxide dismutase; glutathione peroxidase) from symptomatic and asymptomatic cattle with blood parasite infections.

## 2. Materials and Methods

### 2.1. Ethics Statement

This study was conducted according to the guidelines of the Animal Care and Use Committee, Universiti Putra Malaysia, Animal Welfare Act, with approval number, UPM/IACUC/AUP-R008/2020.The cattle owners consented to this study through written consent forms.

### 2.2. Animals and Blood Sample Collection

The selection of cattle for evaluation of serum concentration of cytokines (IL-12, GM-CSF, IFN-γ, IL-10, IL-4) and oxidant/antioxidants (MDA, SOD and GPx) was performed on the basis of physical examination and a PCR analysis of blood samples for parasite detection. The blood samples (6 mL) were obtained from randomly selected 90 crossbred Kedah-Kelantan x Brahman (private farm) and 40 Bali (government farm) cattle breeds of varying ages (1–13 years old) and sex (male and female) via coccygeal venipuncture. These cattle were sampled from private and government beef cattle farms in Muadzam Pahang, Malaysia. The animals were reared in semi-intensive farming system and forages supplemented with minerals. All cattle breeds employed in this study had previous history of blood parasite infections in their respective farms. The clinically healthy cattle were selected based on absence of clinical signs of haemoparasitism (anaemia, icterus, weakness, anorexia, ocular discharge) and absence of tick infestation. Their health status was further confirmed by negative PCR detection of blood parasites, absence of blood parasites on Giemsa-stained blood smear, and normal haematology and serum biochemistry parameters. Blood samples for cytokine and oxidant/antioxidant determinations were placed into plain blood vacutainers while blood for genomic DNA extraction were collected into K2 EDTA blood vacutainers. The blood samples were then transported in an ice box to the haematology and clinical biochemistry laboratory, Universiti Putra Malaysia for processing. Sera was harvested from the clotted blood into microcentrifuge tubes after centrifuging at 2250× *g* for 10 min using portable table centrifuge (EBA 20 Hettich Zentrifugen, Tuttlingen, Germany), and stored at −80 °C until use.

### 2.3. DNA Extraction

Genomic DNA was extracted from K2 EDTA whole blood stored at −80 °C using the DNeasy^®^ Blood and Tissue kit (Qiagen, Hilden, Germany) following the manufacturers’ protocol. The eluted DNA was stored at −20 °C until use. DNA concentration and purity were measured with a Nanodrop spectrophotometer (Tecan Infinite M200^®^, Grodig/Salzburg, Austria GmbH). DNA samples with A_260_/A_280_ ratios between 1.8–2.2 were further analysed.

### 2.4. Detection of Blood Parasites by Conventional PCR

Polymerase chain reaction test was done to amplify the partial gene fragments of *MPSP* gene of *Theileria sinensis*, *MPSP* gene of *Theileria orientalis*, *MSP4* gene of *Anaplasma marginale*, 16S ribosomal RNA gene of *Candidatus Mycoplasma haemobos* and *RoTaT 1.2* VSG of *Trypanosoma evansi* using previously reported species-specific primers. Primers and thermocyclic conditions are presented in Table 1. Forward and reverse primers for *Theileria, sinensis*, *MPSP* gene of *Theileria orientalis*, *MSP4* gene of *Anaplasma marginale*, 16S ribosomal RNA gene of *Candidatus Mycoplasma haemobos* and *RoTaT* 1.2 VSG *Trypanosoma evansi* were as specified by Bai et al. [28], Shkap et al. [29], Ota et al. [30], Urakawa et al. [31] and Su et al. [32] respectively. Each PCR run was performed in a final reaction volume of 25 µL in 0.2 mL PCR reaction tubes, comprising 5× Green GoTaq^®^ flexi buffer (Promega, Madison WI, USA), 25 mM MgCl_2_, 10 mM dNTP mix (dATP, dCTP, dGTP, dTTP), 5 U GoTaq^®^G2 flexi DNA polymerase (Promega), 10 µM of each primer, molecular grade water (Millipore Corporation, Billerica MA, USA) and 100 ng of template DNA. The positive control DNA used in the PCR run consisted of a field isolate confirmed by PCR and sequencing. The field isolate was obtained from the Veterinary Parasitology Laboratory, Universiti Putra Malaysia. For negative control, molecular grade water (Millipore) was substituted for DNA template. PCR amplification was performed using the BioER Little Genius^®^ LED thermal cycler (Hangzhou Bioer Technology, Zhejiang, China). The gel was stained with RedSafe^TM^ (iNtRoN Biotechnology, Jungwon-Gu, Korea) for 10 min and PCR products were examined by 1.2% gel electrophoresis at 80 V for 80 min. The amplified products were visualized using a UV transilluminator (GeneDireX^TM^, Taoyuan, Taiwan).

### 2.5. Quantification of Serum Inflammatory Cytokines in Cattle

Commercial bovine cytokine ELISA quantitation kits were employed to measure interleukin-4 (IL-4) (Bioassay Co., Ltd., Shanghai, China), interferon gamma (Fine Test, Wuhan Fine Biotech Co., Ltd., Wuhan, China), interleukin-10 (Bioassay Co., Ltd.), interleukin 12 (Bioassay Co., Ltd.) and Granulocyte Monocyte-Colony stimulating factor (GM-CSF) (Fine Test, Wuhan Fine Biotech Co., Ltd.), following the manufacturers’ instructions. The absorbance was read at 450 nm with a microplate reader (Infinite 200 pro Tecan, Grodig/Salzburg, Austria) and the serum IL-4, IL-10 and IL-12 values (in ng/L), and GM-CSF and IFN-γ values in (pg/mL) were extrapolated from their respective standard curves. 

### 2.6. Oxidant and Antioxidant Assays

For the oxidant assay, the product of lipid peroxidation (malondialdehyde) was measured in serum by the thiobarbituric acid reactive substances (TBARS) assay as described by Ohkhawa et al. [34] with a slight modification. To determine the serum malondialdehyde concentration, a standard curve was obtained following preparation of standard solutions using 1,1,3,3-tetraethoxypropane (TEP) at different concentrations (0, 0.5, 1, 2.5, 5, 10, 15, 20 MDA/µM). A mixture was obtained by adding 2.4 mL of 1/12 sulphuric acid (H_2_SO_4_) and 0.3 mL of 10% sodium tungstate dehydrate (Na_2_WO_4_) to 300 µl of plasma (or TEP standard or water blank). The mixture is incubated at room temperature for 10 min before being centrifuged using (EBA 20 Hettich Zentrifugen, Tuttlingen, Germany) at 2625× *g* for 10 min. The supernatant was thoroughly removed and 0.05 mL of distilled water, 3 mL of 0.05 M hydrochloric acid (HCL) and 2 µL of 1% thiobarbituric acid (TBA) was added to the sediment to make a reactive mixture. The reactive mixture was made up until 5 mL with distilled water before it was placed in a water bath at 95 °C for 60 min. The boiled mixture was kept at room temperature for 10 min before being centrifuged at 2625× *g* for 10 min. The absorbance of the supernatant was read at 532 nm with a microplate reader (Infinite 200 pro, Tecan) and the results of the serum MDA levels (in nM/mL) were extrapolated from the standard curve.

Serum antioxidant levels were evaluated using commercial ELISA kits to measure superoxide dismutase (SOD) (Elabscience, Houston, TX, USA) by the WST-1 method, and glutathione peroxidase (GPx) (Bioassay Technology Laboratory, Shanghai Korain Biotech Co., Ltd., Shanghai, China) following methods described in the kits. The optical density was read at 450 nm with a microplate reader (Infinite 200 pro, Tecan), and results of serum SOD and GPx were extrapolated from their respective standard curves.

### 2.7. Statistical Analysis

Data analysis was performed using SPSS 25.0 (Chicago, IL, USA) following normal distribution of data (Shapiro-Wilk’s test). Data were expressed as mean ± standard error. Values for all the parameters measured for each cattle group were extrapolated from their standard curves respectively. 95% Confidence interval was calculated using proportion test https://epitools.ausvet.com.au/ciproportion (accessed on 12 November 2020). One way analysis of variance (ANOVA) followed by Duncan multiple post hoc comparison test was applied on all data generated from this study. Only the data generated from single blood parasite infected cattle and clinically healthy cattle were utilized in this study. Cattle infected with more than one blood parasite were not involved in this study. Pearson’s correlation coefficient was used for determining the association between factors (age, gender and clinical symptoms) and cytokine level in each of the blood parasite infected cattle. *p* < 0.05 was considered statistically significant. Determination of the effect of age and farm location on the oxidative stress parameters were done using independent sample t-test. The differences in the response to infection and oxidative stress with respect to age and farm ownership were determined by independent sample t-test. *p* < 0.05 was considered as statistically significant.

## 3. Results

### 3.1. Molecular Detection Rate of Blood Parasites in the Symptomatic and Asymptomatic Cattle

The following blood parasite species were detected by PCR in both symptomatic and asymptomatic cattle: *Theileria orientalis, Theileria sinensis, Candidatus Mycoplasma haemobos, Anaplasma marginale* and *Anaplasma platys*. *Trypanosoma evansi* was only detected in the crossbred Kedah-Kelantan x Brahman cattle. Non-specific clinical signs such as weakness, pallor of mucous membrane, inappetence, cachexia, lateral recumbency, icterus, dehydration, mild tick infestation was only observed in most of the symptomatic cattle.

The detection rates of blood parasites in this study are presented in Table 2. *Candidatus Mycoplasma haemobos* was the blood parasite with the highest detection rate 70/130 (53.9%; 95% CI 45.3–62.2%), followed by *Theileria orientalis* 65/130 (50%; 95% CI 41.5–58.5%), *A. marginale* 49/130 (37.8%; 95% CI 29.8–46.3%), *T. sinensis* 32/130 (24.6%; 95% CI 18.0–32.3%), *A. platys* 7/130; (5.38%; 95% CI 2.63–10.7%) and *Trypanosoma evansi* 4/130 (3.08%; 95 CI 1.20–7.64%) (Table 1). About 47/130 (36.25%) cattle had single blood parasite infection while 63/130 (48.5%) were infected with more than one blood parasite species.

### 3.2. Serum Proinflammatory, Antiinflammatory Cytokine and Oxidant/Antioxidant Levels in Symptomatic and Asymptomatic Cattle

#### 3.2.1. Serum Cytokine and Oxidant/Antioxidant Levels in Symptomatic and Asymptomatic Cattle Naturally Infected with *Candidatus Mycoplasma haemobos*

The results of the serum cytokine and oxidant/antioxidant levels in symptomatic and asymptomatic cattle naturally infected with *Candidatus Mycoplasma haemobos* were presented in Table 3. The serum level of IL-12 in *C. M. haemobos*-infected cattle (asymptomatic cattle) tended to be the highest when compared with that of the *C. M. haemobos*-infected cattle (symptomatic cattle) groups and clinically healthy cattle (*p* < 0.05), with 1.39 and 2.42-fold increases in serum IL-12 levels compared to *C. M. haemobos*-infected cattle (symptomatic cattle) and clinically healthy cattle (*p* < 0.05). Also, the mean IL-12 value of *C. M. haemobos*-infected cattle (symptomatic cattle) was significantly higher (*p* < 0.05) than that of clinically healthy cattle (Table 3). The serum IL-10 level of the *C. M. haemobos*-infected cattle (asymptomatic cattle) was higher than those of the other cattle groups (*p* < 0.05), with a 1.24-fold and 2.58-fold increase compared to *C. M. haemobos* infected cattle (symptomatic cattle) and clinically healthy cattle, respectively. Both symptomatic and asymptomatic *Candidatus Mycoplasma haemobos*-infected cattle had the highest mean GM-CSF value (*p* < 0.05) with 9.13 and 8.79-fold increases, respectively, in their serum GM-CSF level when compared to clinically healthy cattle. The level of IFN-γ in the serum of *C. M. haemobos* infected cattle (asymptomatic cattle) increased by 1.13 and 1.10-fold, relative to *C. M. haemobos* infected cattle (symptomatic cattle) and clinically healthy cattle (*p* < 0.05). A significant increase in the serum level of IL-4 was recorded in both symptomatic and asymptomatic *C. M. haemobos*-infected cattle, relative to clinically healthy cattle (*p* < 0.05). There was no correlation between other factors (age and gender) and cytokine level in the *C. Mycoplasma haemobos* (*p* < 0.05)-infected cattle. However, a negative correlation existed between clinical symptoms and cytokines: IL-12 (*r* = −0.685; *p* = 0.001), IL-10 (*r* = −0.618; *p* = 0.003), GM-CSF (*r* = −0.315; *p* = 0.006) and IL-4 (*r* = −0.409; *p* = 0.000) (Table S1).

The MDA level in sera from both symptomatic and symptomatic *C. M. haemobos*-infected cattle was significantly higher than that from serum of clinically healthy cattle (*p* < 0.05). Also, high serum activities of SOD and GPx were recorded in both symptomatic and asymptomatic cattle infected with *C. M. haemobos,* and were significantly higher than that of clinically healthy cattle, with the mean SOD value of the *C. M. haemobos*-infected cattle (asymptomatic) being highest among the cattle groups (Table 3). In addition, the data showed a 1.8-fold increase in the serum SOD activity of *C. M. haemobos*-infected cattle (symptomatic), relative to the clinically healthy cattle, and a 1.03-fold increase compared to *C. M. haemobos*-infected cattle (asymptomatic). Increased serum GPx activity was found in both symptomatic and asymptomatic *C. M. haemobos*-infected cattle, with a 2.54-fold increase in their GPx value compared to clinically healthy cattle group (*p* < 0.05) (Table 3). The effect of farm ownership (private and government) and age on the oxidant and antioxidative stress responses was determined too and the serum SOD activity of *C. M. haemobos* cattle sampled from private farm (941.85 ± 15.52) was significantly higher than that from cattle sampled from government farm (876.49 ± 21.35), (*p* = 0.017). However, age had no significant effect on the oxidative and anti-oxidative stress responses in *C. M. haemobos*-infected cattle (*p* > 0.05) (Table S7).

#### 3.2.2. Serum Cytokine Levels and Oxidant/Antioxidant Levels in Symptomatic and Asymptomatic Cattle Naturally Infected by *Theileria orientalis*

The results from cattle infected with *Theileria orientalis* (symptomatic and asymptomatic) revealed high serum levels of IL-12 and IL-10 compared to clinically healthy cattle (*p* < 0.05) (Table 4). Specifically, 2.47-fold and 2.35-fold increases in mean IL-12 and IL-10 values were found in *Theileria orientalis*-infected cattle (symptomatic), relative to clinically healthy cattle (*p* < 0.05). However, no significant differences were found in the mean IL-12 and IL-10 values of symptomatic *Theileria orientalis* and asymptomatic *Theileria orientalis*- infected cattle (*p* > 0.05). Increased serum GM-CSF value were recorded in the symptomatic and asymptomatic *Theileria orientalis*-infected cattle l (*p* < 0.05), with a 5.57 and 5.80-fold increases in their GM-CSF value when compared to clinically healthy cattle. In addition, increases in serum IL-4 levels were found in both symptomatic (1.97-fold) and asymptomatic (2.02-fold) *Theileria orientalis*-infected cattle, relative to the clinically healthy cattle (*p* < 0.05). Significant increase in mean IFN-γ value was recorded for both symptomatic and asymptomatic *T. orientalis*-infected cattle, with 1.7 and 1.6-fold increases in their mean IFN-γ values compared to clinically healthy cattle (Table 4). A Pearson’s correlation coefficient test revealed a negative correlation between gender and IL-10 (*r* = −0.294; *p* = 0.022) in the *T. orientalis*-infected cattle. In addition, a negative correlation was found between clinical symptoms and cytokine level [IL-12 (*r* = −0.323; *p* = 0.007), IL-10 (*r* = −0.374; *p* = 0.002), GM-CSF (*r* = −0.279; *p* = 0.007), IL-4 (*r* = −0.406; *p* = 0.001) and IFN-γ (*r* = −0.337; *p* = 0.005)] of *T. orientalis*-infected cattle (Table S2).

The mean MDA values of *Theileria orientalis*-infected cattle (symptomatic and asymptomatic) were significantly higher than those of the clinically healthy cattle (*p* < 0.05), with a 2.2−2.9—fold increases, relative to the latter. Also, mean SOD and GPx values of both the symptomatic and asymptomatic *Theileria orientalis*-infected cattle were higher than that of the clinically healthy cattle (*p* < 0.05), with the most significant increase in mean SOD value observed in the *Theileria orientalis*-infected cattle (asymptomatic) showing a 1.7-fold increase in its serum SOD activity when compared to the clinically healthy cattle. However, no significant difference in the mean GPx values was observed between the symptomatic and asymptomatic cattle infected with *Theileria orientalis* (*p* > 0.05), but the serum activity was significantly higher than that of clinically healthy cattle (*p* < 0.05) (Table 4). There were no significant differences in the oxidative and antioxidative stress responses from *T. orientalis*-infected cattle sampled from private and government farms (*p* > 0.05. Age was not computed because all the *T. orientalis*-infected cattle were adults (8–13 years) (Table S8).

#### 3.2.3. Serum Cytokine and Oxidant/Antioxidant Levels in Symptomatic and Asymptomatic Cattle Naturally Infected with *Anaplasma marginale*

In symptomatic and asymptomatic cattle naturally infected with *Anaplasma marginale*, an increase in mean IL-12 and IL-10 values were recorded in comparison to clinically healthy cattle (*p* < 0.05). The asymptomatic *A. marginale*-infected cattle had the highest mean IL-12 and IL-10 values, with 1.9-fold and 15.8-fold increases in serum IL-12 level, and 8.7-fold and 17.5-fold increases in serum IL-10 level, relative to symptomatic *A. marginale*-infected cattle and clinically healthy cattle, respectively. A 1.0-fold and 6.3-fold increase in serum GM-CSF level were found in symptomatic *A. marginale*-infected cattle compared to asymptomatic cattle and clinically healthy cattle, respectively (*p* < 0.05). The highest mean IL-4 value was recorded for the asymptomatic *A. marginale*-infected cattle, whose IL-4 value was higher by 1.3-fold and 2.8-fold, relative to symptomatic *A. marginale*- infected cattle and clinically healthy cattle (*p* < 0.05). The level of IFN-γ was also higher in sera from symptomatic and asymptomatic cattle infected with *A. marginale,* compared with the clinically healthy cattle. In addition, a 1.3-fold increase in serum IFN-γ level was found in asymptomatic *A. marginale*-infected cattle compared to symptomatic *A. marginale* infected cattle, and 2.2-fold increase compared to clinically healthy cattle (Table 5). No correlation (*p* > 0.05) was found between gender and cytokine level, for the except IL-12 value (*r* = 0.300; *p* = 0.051) which was positively correlated with gender and nearly significant (Table S3). Also, a negative correlation existed between clinical symptoms and cytokine level [IL-12 (*r* = −0.574; *p* = 0.000), IL-10 (*r* = −0.507; *p* = 0.000), GM-CSF (*r* = −0.426; *p* = 0.002), IL-4 (*r* = −0.541; *p* = 0.000) and IFN-γ (*r* = −0.524; *p* = 0.000)] of *A. marginale*-infected cattle (Table S3).

In both symptomatic and asymptomatic *A. marginale*-infected cattle, a significantly higher serum level of MDA, and higher serum activities of SOD and GPx were recorded, relative to the clinically healthy cattle (*p* < 0.05). However, there were no significant difference in the mean SOD value of both symptomatic and asymptomatic *A. marginale*-infected cattle. In addition, 2.3 and 1.7-fold increases in serum SOD activities were found in *A. marginale*-infected cattle (symptomatic) and *A. marginale*-infected cattle (asymptomatic) respectively, compared to the clinically healthy cattle (*p* < 0.05). A 2.7-fold and 5.1-fold increases in serum GPx activities were found in *A. marginale*-infected cattle (symptomatic) and *A. marginale* (asymptomatic) respectively, compared to the clinically healthy cattle (*p* < 0.05) (Table 5). A significantly higher serum SOD activity was recorded in the *A. marginale*-infected cattle sampled from private farm (989.61 ± 12.90), relative to those from the government farm (844.98 ± 16.28, *p* = 0.003) (Table S8). The effect of age was not computed because all *A. marginale* infected cattle were adults (8–13 years) (Table S9).

#### 3.2.4. Serum Cytokine Levels in Symptomatic and Asymptomatic Cattle Naturally Infected with *Theileria sinensis*

The results of the serum levels of cytokine and oxidant/antioxidant in cattle infected with *Theileria sinensis* are presented in Table 6. High IL-12 and IL-10 level were found in serum from symptomatic cattle infected with *T. sinensis*, relative to asymptomatic cattle infected with *T. sinensis* and clinically healthy cattle (*p* < 0.05), but, no significant difference in the mean IL-12 value was found in *T. sinensis*-infected cattle (asymptomatic) and clinically healthy cattle (*p* > 0.05). In addition, 5.1-fold and 1.3-fold increases in the serum IL-12 and IL-10 level, respectively, were found in symptomatic *T. sinensis*-infected cattle, relative to asymptomatic *T. sinensis*-infected cattle. Significant increases in the mean values of GM-CSF was recorded from the serum of both symptomatic (4.2-fold) and asymptomatic (3.9-fold) *T. sinensis*-infected cattle, compared to clinically healthy cattle. Also, when compared to the clinically healthy cattle, the symptomatic and asymptomatic *T. sinensis*- infected cattle had a higher serum level of IL-4 (*p* < 0.05). Specifically, a 2.8 and 2.0-fold increase in the mean IL-4 value was recorded for the symptomatic *T. sinensis*-infected cattle and asymptomatic *T. sinensis*-infected cattle, respectively, compared to the clinically healthy cattle. The IFN-γ level from the serum of symptomatic *T. sinensis*-infected cattle was higher than that of the asymptomatic *T. sinensis*-infected cattle, and their mean IFN-γ values were significantly higher relative to clinically healthy cattle (*p* < 0.05). Furthermore, the mean IFN-γ value increased significantly by 2.1-fold in the symptomatic *T. sinensis*-infected cattle and by 1.5-fold increase in the asymptomatic *T. sinenis*-infected cattle when compared to the clinically healthy cattle. The Pearson’s correlation coefficient revealed a negative correlation between clinical symptoms and cytokine level [(IL-12 (*r* = −0.444; *p* = 0.005), IL-10 (*r* = −0.433; *p* = 0.006), GM-CSF (−0.600; *p* = 0.000), IL-4 (*r* = −0.488; *p* = 0.002) and IFN-γ (*r* = −0.441; *p* = 0.005). However, IL-4 was the only cytokine found to be negatively correlated with age (*r* = −0.512; *p* = 0.003) There was no correlation (*p* > 0.05) between gender and cytokine level (Table S4).

The mean MDA value was increased significantly in the asymptomatic *T. sinensis*-infected cattle by 2.9-fold and by 3.7-fold in the symptomatic *T. sinensis*-infected cattle when compared to the clinically healthy cattle. Both symptomatic and asymptomatic cattle infected with *T. sinensis* all showed significant increases in their mean SOD and GPx values when compared to the clinically healthy cattle (*p* < 0.05). Furthermore, a 1.8-fold increase in mean SOD values was recorded for both symptomatic and asymptomatic *T. sinensis*-infected cattle, respectively, in comparison to clinically healthy cattle. Also, when compared to clinically healthy cattle, a 4.5-fold increase and 4.4-fold increase in mean GPx value were also recorded for symptomatic *T. sinensis* and asymptomatic *T. sinensis*-infected cattle respectively (Table 6). There was no significant effect of farm ownership (private and government) and age (young and adult) on the oxidative and anti-oxidative stress responses of *T. sinensis* infected cattle (*p* > 0.05) (Table S10).

#### 3.2.5. Serum Cytokine and Oxidant/Antioxidant Levels in Symptomatic Cattle Naturally Infected with *Trypanosoma evansi*

The results of the serum cytokine and oxidant/antioxidant levels in cattle infected with *Trypanosoma evansi* are presented in Table 7. The mean IL-12 and IL-10 values increased, respectively, in the symptomatic *Trypanosoma evansi*-infected cattle by 3.8-fold and 3.6-fold, relative to clinically healthy cattle (*p* < 0.05). A similar trend was observed for the serum GM-CSF and IL-4 levels in the symptomatic *T. evansi*-infected cattle, with 6.8-fold and 1.7-fold increases in the serum GM-CSF and IL-4, respectively, compared to the clinically healthy cattle (*p* < 0.05). Also, the serum level of IFN-γ in the *T. evansi*-infected cattle was higher than that of the clinically healthy cattle by a 1.7-fold increase (*p* < 0.05). The Pearson’s correlation coefficient test revealed a negative correlation between clinical symptoms and cytokine level [ IL-12 (*r* = −0.984; *p* = 0.000), IL-10 (*r* = −0.996; *p* = 0.000), GM-CSF (*r* = −0.996; *p* = 0.000), IL-4 (*r* = −0.997; *p* = 0.000) and IFN-γ (*r* = −0.978; *p* = 0.000) in the *T. evansi*-infected cattle (Table S5). The MDA level was significantly higher in serum from symptomatic *T. evansi,* compared to serum from clinically healthy cattle (*p* < 0.05). Also, high serum activities of SOD and GPx were found in symptomatic *T. evansi* cattle when compared to the clinically healthy cattle. The data showed a 2.1-fold increase in the serum SOD activity of *T. evansi* -infected cattle relative to the clinically healthy cattle. Furthermore, 2.4-fold increase in serum GPx activity was found in *T. evansi*-infected cattle relative to clinically healthy cattle (*p* < 0.05).

#### 3.2.6. Serum Cytokine Levels and Oxidant/Antioxidant Levels in Symptomatic Cattle Naturally Infected with *Anaplasma platys*

A 24.8-fold increase in serum IL-12 level was found in symptomatic *A. platys*-infected cattle compared to clinically healthy cattle (*p* < 0.05). Likewise, the serum IL-10 level was highest in the symptomatic *A. platys*-infected cattle, with a 35.1-fold increase compared to clinically healthy cattle (*p* < 0.05). Furthermore, GM-CSF value obtained from serum of symptomatic *A. platys*-infected cattle was significantly higher than GM-CSF value obtained from sera of clinically healthy cattle by a 5.2-fold increase (*p* < 0.05). The mean IL-4 value of symptomatic *A. platys* was 3.7-fold higher than that of clinically healthy cattle (*p* < 0.05). Also, the mean IFN-γ value of symptomatic *A. platys*-infected cattle was 2.1-fold higher than that of clinically healthy cattle (*p* < 0.05) (Table 8). There was no correlation between gender and cytokine level in the *A. platys*-infected cattle. However, a negative correlation existed between clinical symptoms and cytokine level [IL-12 (*r* = −0.785; *p* = 0.002), IL-10 (*r* = −0.670; *p* = 0.005), GM-CSF (*r* = −0.941; *p* = 0.000) (Table S6). The mean MDA value of the symptomatic *A. platys*-infected cattle was significantly higher than that of the clinically healthy cattle (*p* < 0.05), with a 4.3-fold increase. Also, serum SOD and GPx activities of symptomatic *A. platys*-infected cattle were higher than those of the clinically healthy cattle (*p* < 0.05), showing 2.9 and 6.3-fold increases in the serum SOD and GPx activities, respectively, relative to the clinically healthy cattle (Table 8). *Anaplasma platys*-infected cattle from the private farm (1561.46 ± 15.74) had a significantly higher serum SOD activity relative to those from the government farm (1029.26 ± 13.14) (*p* = 0.033). The effect of age is unknown as all the *A. platys* infected cattle were all adults (Table S11).

### 3.3. Pro-Inflammatory and Anti-Inflammatory Cytokine Ratios in Symptomatic and Asymptomatic Cattle Naturally Infected with Blood Parasites

#### 3.3.1. *Candidatus Mycoplasma haemobos*-Infected Cattle

**Figure 1:**
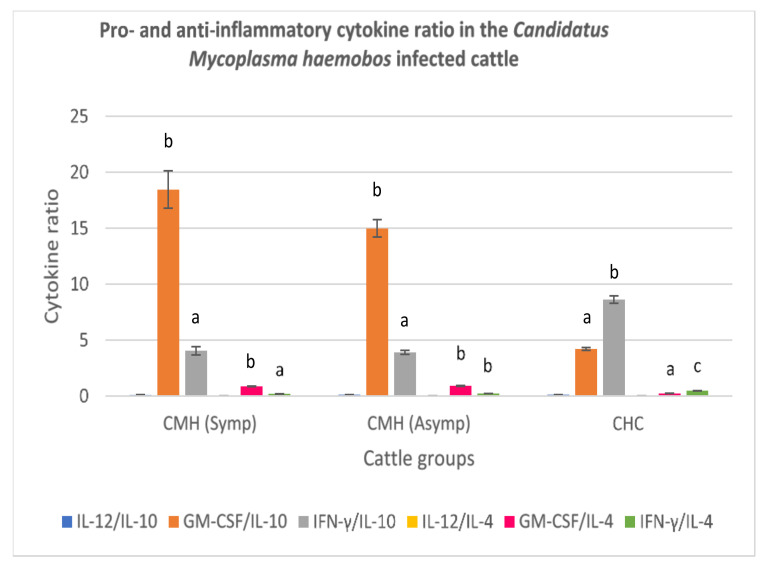
Pro- and anti-inflammatory cytokine ratio in symptomatic and asymptomatic cattle naturally infected with *Candidatus Mycoplasma haemobos.* Molecular detection and confirmation of the blood parasite was by PCR amplification of 16S rRNA gene. *Candidatus Mycoplasma haemobos* infected symptomatic cattle had the highest serum GM-CSF:IL-10 cytokine ratio. Low IL-12:IL-10, IL-12:IL-4, IFN-γ:IL4 ratios were significant findings in both symptomatic and asymptomatic *C. M. haemobos* infected cattle. CMH (Symp): Symptomatic *Candidatus Mycoplasma haemobos* infected cattle; CMH (Asymp): Asymptomatic *Candidatus Mycoplasma haemobos* infected cattle; CHC: Clinically healthy cattle (control). IL-12: Interleukin 12; IL-10: Interleukin 10; GM-CSF: Granulocyte Macrophage-colony stimulating factor; IFN-γ: Interferon gamma; IL-4: Interleukin 4. (**a**–**c**) Different superscripts refer to significant differences in the parameters between *C. M. haemobos* infected cattle and clinically healthy cattle (*p* < 0.05).

The balance of pro- and anti-inflammatory cytokines reflected by the cytokine ratios were determined for the symptomatic and asymptomatic cattle naturally infected by *C. M. haemobos,* and the clinically healthy cattle group. A very low IL-12:IL-10 ratio was recorded for the symptomatic and asymptomatic *C. M. haemobos*-infected cattle, and their cytokine ratios did not differ significantly (*p* > 0.05) from those of the clinically healthy cattle (Figure 1). A high GM-CSF to IL-10 ratio was recorded for the symptomatic and asymptomatic *C. M. haemobos*-infected cattle, relative to clinically healthy cattle. A very low IFN-γ:IL-10 ratio was recorded for both symptomatic and asymptomatic *C. M. haemobos*-infected cattle. Also, a very low IL-12:IL-4 was recorded for both symptomatic and asymptomatic *C. M. haemobos*-infected cattle. However, there were no significant difference in the IL-12:IL-4 ratio of all the cattle groups (*p* > 0.05). Also, a slightly high GMCSF:IL-4 was recorded from the serum of symptomatic and asymptomatic *C. M. haemobos*-infected cattle, relative to the clinically healthy cattle. Likewise, a very low IFN-y:IL-4 ratio was recorded for the symptomatic and asymptomatic *C. M. haemobos*-infected cattle when compared with the ratio obtained from sera of clinically healthy cattle (*p* < 0.05) (Figure 1).

#### 3.3.2. *Theileria orientalis*-Infected Cattle

**Figure 2:**
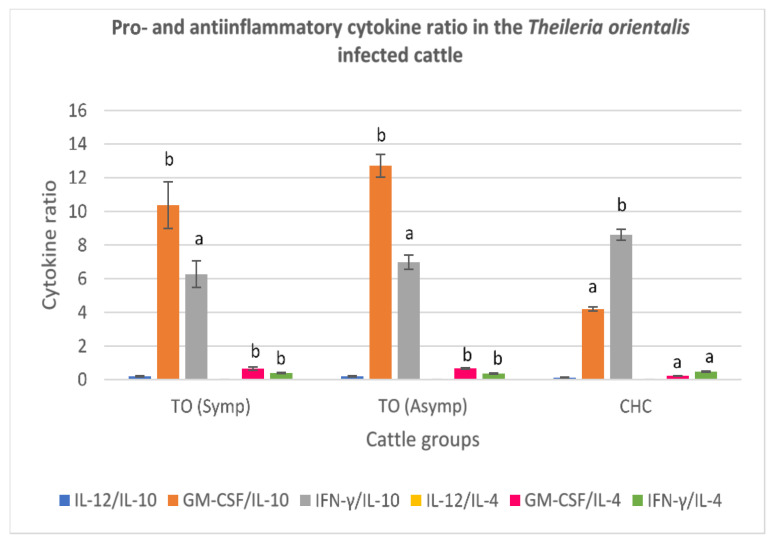
Pro- and anti-inflammatory cytokine ratio in symptomatic and asymptomatic cattle infected by *Theileria orientlalis*. Molecular detection and confirmation of *T*. *orientalis* was by PCR amplification of the MPSP gene. High serum GM-CSF:IL-10 and GM-CSF:IL-4, and low IL-12:IL-10 levels were observed in both symptomatic and asymptomatic cattle infected with *Theileria orientalis.* Also, low IL-12:IL-4 and IFN-γ:IL-10 levels were found in both symptomatic and asymptomatic *T. orientalis* infected cattle. TO (Symp): Symptomatic *Theileria orientalis* infected cattle. TO (Asymp): Asymptomatic *Theileria orientalis* infected cattle. CHC: Clinically healthy cattle (control). IL-12: Interleukin 12; IL-10: Interleukin 10; GM-CSF: Granulocyte Macrophage-colony stimulating factor; IFN-γ: Interferon gamma; IL-4: Interleukin 4. (**a**,**b**) Different superscripts refer to significant differences in the parameters between *T. orientalis* infected cattle and clinically healthy cattle (*p* < 0.05).

A low serum IL-12:IL-10 ratio was recorded from both the symptomatic and asymptomatic *T. orientalis*-infected cattle. (Figure 2). Also, a high GM-CSF:IL-10 ratio was recorded for the symptomatic and asymptomatic *T. orientalis*-infected cattle, compared to clinically healthy cattle (*p* < 0.05). The serum IFN-y:IL:10 and IL-12:IL-4 ratios were also low in the symptomatic and asymptomatic *T. orientalis*-infected cattle. A high GM-CSF:IL-4 was reported for both symptomatic and asymptomatic *T. orientalis*-infected cattle groups, relative to clinically healthy cattle (Figure 2). Furthermore, a low IFN-γ:IL-4 value was obtained for the symptomatic and asymptomatic *T. orientalis*-infected cattle, relative to the clinically healthy cattle (*p* < 0.05) (Figure 2).

#### 3.3.3. *Anaplasma marginale*-Infected Cattle

**Figure 3:**
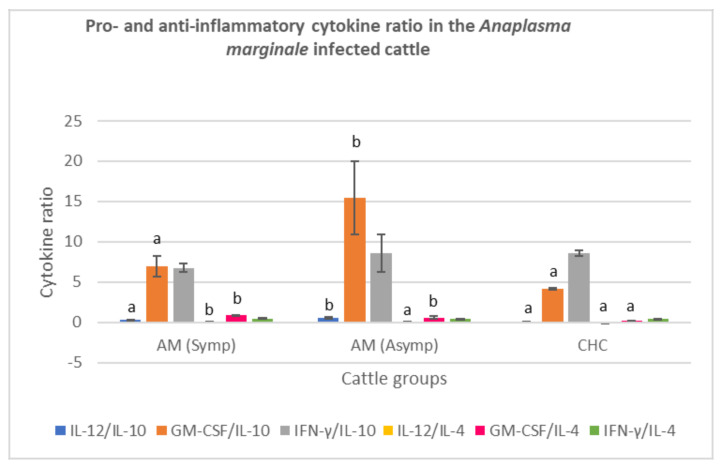
Pro- and anti-inflammatory cytokine ratio in symptomatic and asymptomatic cattle infected with *A. marginale.* Molecular detection and confirmation of *A. marginale* was by PCR amplification of the MSP4 gene. High serum GM-CSF:IL-4 level was observed in both symptomatic and asymptomatic *A. marginale* infected cattle. AM (Symp): Symptomatic *Anaplasma marginale* infected cattle, AM (Asymp): Asymptomatic *Anaplasma marginale* infected cattle. CHC: Clinically healthy cattle (control): IL-12: Interleukin 12; IL-10: Interleukin 10; GM-CSF: Granulocyte Macrophage-colony stimulating factor; IFN-γ: Interferon gamma; IL-4: Interleukin 4. (**a**,**b**) Different superscripts refer to significant differences in the parameters between *A. marginale* infected cattle and clinically healthy cattle (*p* < 0.05).

The ratio of IL-12 to IL-10 obtained from the serum of asymptomatic *A. marginale*- infected cattle was high (*p* < 0.05), however no significant difference in the IL-12:IL-10 ratio between the symptomatic *A. marginale* infected cattle and clinically healthy cattle were recorded (*p* > 0.05). A very high serum GM-CSF:IL-10 ratio was also recorded for asymptomatic *A. marginale* infected cattle relative to symptomatic *A. marginale* infected cattle and clinically healthy cattle. However, there was no significant difference in the mean IFN-γ:IL-10 ratio of all cattle groups (*p* > 0.05). A very low IL-12:IL-4 ratio was obtained from asymptomatic *A. marginale* = infected cattle compared to clinically healthy cattle. Also, a high GM-CSF:IL-4 ratio was recorded for both symptomatic and asymptomatic *A. marginale*-infected cattle, relative to clinically healthy cattle (*p* < 0.05). However, IFN-γ:IL-4 value of the cattle groups did not differ from each other (*p* > 0.05) (Figure 3).

#### 3.3.4. *Theileria sinensis*-Infected Cattle

**Figure 4:**
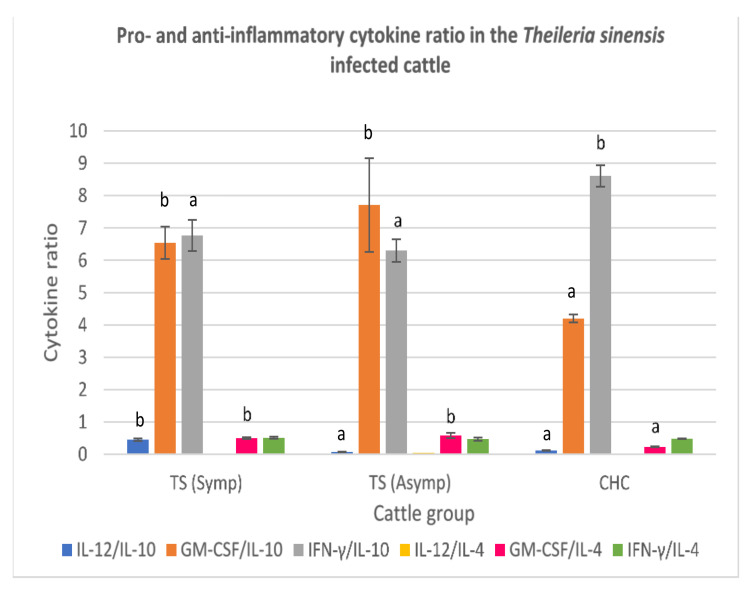
Pro- and anti-inflammatory cytokine ratio in symptomatic and asymptomatic cattle infected with Theileria sinensis. Molecular detection and confirmation of blood pathogens were by PCR ampli-fication of MPSP gene. High serum GM-CSF: IL-10 and GM-CSF:IL-4 ratios were observed in both symptomatic and asymptomatic Theileria sinensis infected cattle, respectively. TS (Symp): Symp-tomatic Theileria sinensis infected cattle. TS (Asymp): Asymptomatic Theileria sinensis infected cattle. CHC: Clinically healthy cattle. (control). IL-12: Interleukin 12; IL-10: Interleukin 10; GM-CSF: Granulocyte Macrophage-colony stimulating factor; IFN-γ: Interferon gamma; IL-4: Interleukin 4. (**a**,**b**) Different superscripts refer to significant differences in the parameters between T. sinensis infected cattle and clinically healthy cattle (*p* < 0.05).

In the symptomatic cattle infected with *Theileria sinensis,* high IL-12:IL-10 was recorded relative to asymptomatic *Theileria sinensis*-infected cattle and clinically healthy cattle (*p* < 0.05). A high GM-CSF:IL-10 was recorded for the symptomatic and asymptomatic *T. sinensis*-infected cattle. There was no significant difference in the IFN-γ:IL-10 ratio of both symptomatic and asymptomatic *T. sinensis*-infected cattle (*p* > 0.05), but their IFN-γ:IL-10 was lower than that of clinically healthy cattle. The serum IL-12:IL-4 ratio from the symptomatic *T. sinensis*-infected cattle was lower than that obtained from the sera of asymptomatic *T. sinensis*-infected cattle and clinically healthy cattle (*p* < 0.05). High GM-CSF:IL-4 ratio was recorded for both symptomatic and asymptomatic *T. sinensis*-infected cattle, relative to clinically healthy cattle. The serum IFN-γ:IL-4 ratio was low in all cattle groups but did not differ significantly from each other (*p* > 0.05) (Figure 4).

#### 3.3.5. *Trypanosoma evansi*-Infected Cattle

**Figure 5:**
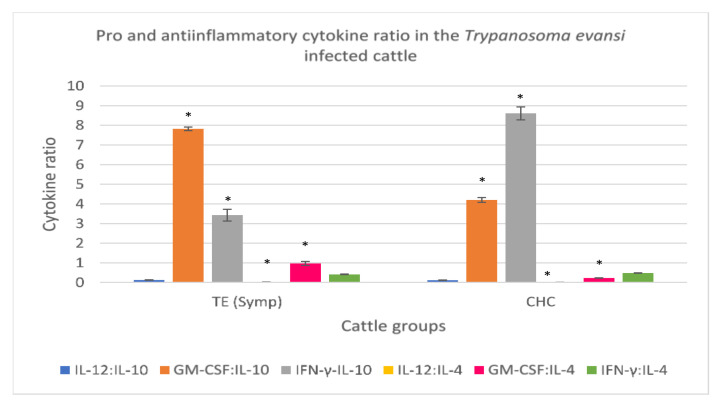
Pro- and anti-inflammatory cytokine ratio in symptomatic cattle naturally infected with Table 1. 2 VSG gene. High serum GM-CSF:IL-10 and GM-CSF:IL-10 ratios, and low IL-12:IL-10 and IFN-γ:IL-10 ratio were prominent findings in the *Trypanosoma evansi* infected cattle. CHC: Clinically healthy cattle (control). IL-12: Interleukin 12; IL-10: Interleukin 10; GM-CSF: Granulocyte Macrophage-colony stimulating factor; IFN-γ: Interferon gamma; IL-4: Interleukin 4. * Asterisk superscript on a parameter indicates significant difference *p* < 0.05.

To determine the balance of pro- and anti-inflammatory cytokines in clinical trypanosomosis, cytokine ratios were determined in the *T. evansi*-infected cattle and clinically healthy cattle. A low IL-12:IL-10 ratio was recorded for the *T. evansi*-infected cattle and clinically healthy cattle though not significant. High GM-CSF:IL-10 and GM-CSF:IL-4 and low IFN-γ:IL-10 were recorded in the *T. evansi*-infected cattle when compared to clinically healthy cattle. A slight increase in IL-12:IL-4 was also recorded in the *T. evansi*-infected cattle when compared to clinically healthy cattle. IFN-γ:IL-4 ratio in the serum from *T. evansi*-infected cattle was low but did not differ from that of clinically healthy cattle (Figure 5).

#### 3.3.6. *Anaplasma platys*-Infected Cattle

**Figure 6:**
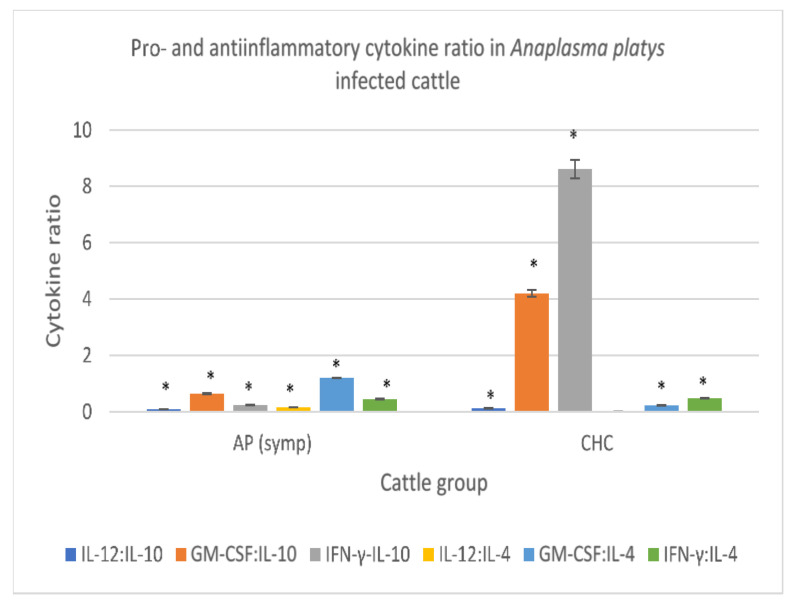
Pro- and anti-inflammatory cytokine ratio in symptomatic cattle infected with *Anaplasma platys*. Molecular detection and confirmation of *Anaplasma platys* was by PCR amplification of the 16S rRNA gene using species-specific primers. Low serum IL-12:IL-10, GM-CSF:IL-10 and IFN-y:IL-10, and high GM-CSF:IL-4 ratio was observed in the symptomatic cattle infected with *Anaplasma platys*. AP (Symp): Symptomatic *Anaplasma platys* infected cattle. CHC: Clinically healthy cattle (control). IL-12: Interleukin 12; IL-10: Interleukin 10; GM-CSF: Granulocyte Macrophage-colony stimulating factor; IFN-γ: Interferon gamma; IL-4: Interleukin 4. * Asterisk superscript on a parameter indicates significant difference *p* < 0.05.

In the symptomatic cattle infected with *A. platys,* a low IL-12:IL-10, GM-CSF:IL-10 and IFN-γ:IL-10, relative to clinically healthy cattle, were recorded. A high IL-12:IL-4 ratio was also recorded in the *A. platys*-infected cattle showing clinical signs of the disease. Likewise, a high serum GM-CSF:IL-4 ratio was found in symptomatic *A. platys*-infected cattle, and the IFN-γ:IL-4 value obtained from serum of symptomatic *A. platys*-infected cattle which did not differ significantly from that of clinically healthy cattle were also recorded (*p* > 0.05) (Figure 6).

## 4. Discussion

This study investigated immune and oxidant/antioxidant responses concerning pro- and anti-inflammatory cytokine levels and oxidant/antioxidant levels in cattle naturally infected by different blood parasites, respectively. In many diseases, a balance between pro-inflammatory/anti-inflammatory cytokines has been reported to be an important marker of infection and disease outcomes. The balance in these cytokine levels has been demonstrated in infectious [35] and non-infectious diseases [36], and their activities are highly dependent upon their serum concentration and duration of expression [23]. The present study showed that *A. marginale, T. orientalis, T. evansi, A. platys* and *T. sinensis* infected cattle had increased level of serum IL-12 and IFN-γ. Interleukin 12 and IFN- γ (proinflammatory cytokines) are involved in blood parasite clearance and cytokine mediated pathology. The over expression of these pro-inflammatory cytokines could have exacerbated the severity of these vector-borne diseases, but IL-10 and IL-4 inhibited the pro-inflammatory cytokine activity, evidenced by low IL-12/IL-10 ratio, low IFN-γ/IL-10 and low IFN-γ/IL-4 ratios reported in these cattle groups. However, it seems there is an immunomodulatory effect, as the massive production of IL-12 and IFN-γ was rapidly regulated by IL-10 and IL-4. The IL-10 and IL-4, therefore, might have attenuated or resolved the inflammatory response. The finding of a low IL-12/IL-10 cytokine ratio in *T. evansi* agreed with Bakari et al. [6], who reported a decreasing trend of IL-12 and a sustained IL-10 cytokine in cattle naturally infected by *Trypanosoma species*. Suppression of IL-12 and IFN-γ activities however, results in reduced immunopathology, tissue destruction and multiple organ failure observed in natural blood parasite infection in cattle [37,38]. Interferon-gamma was one of the cytokines detected in high amounts in sera from both symptomatic and asymptomatic cattle. Our findings suggest that IFN-γ might be one of the cytokines that persists longer in circulation after resolution of the primary infection or it is among the pro-inflammatory cytokines such as IL-6 that has the ability to persist longer in circulation in animals manifesting the clinical signs of blood parasite infection and asymptomatic carrier cattle [39].

The finding of an anti-inflammatory immune response characterized by low IL-12:IL-10 ratio in symptomatic *Theileria orientalis*-infected cattle groups, was however, in contrast with *Theileria annulata* infection in Egyptian cattle, where pro-inflammatory cytokine response was observed [23]. This difference could be due to the stage of infection, as pro-inflammatory cytokine responses are usually prominent in the early stage of an infection [40] and we could not ascertain the time origin of infection because this study was a field one. The pro-inflammatory cytokine immune response in the asymptomatic *T. orientalis* cattle characterized by high IL-12/IL-10 and high IL-12/IL-4 ratios, and symptomatic *A. marginale* infected cattle characterized by high IL-12/IL-10 ratio thus signifies close contact of the susceptible host with their respective vectors in the farmhouse, persistent antigenic stimulation or the animals may be recovering from the disease.

Granulocyte macrophage-colony stimulating factor was found to be present in high amounts in the serum of all blood parasite infected cattle groups with or without the clinical signs of the disease, but the predominant GM-CSF pro-inflammatory cytokine immune response characterized by high GM-CSF:IL-10 ratio were present in the symptomatic and asymptomatic *T. orientalis* and *C. Mycoplasma haemobos,* symptomatic *T. evansi,* and symptomatic and asymptomatic *T. sinensis*-infected cattle. This indicates a possible response to low white blood cell count, especially low neutrophil count, in the blood parasite-infected cattle reported in our previous study [4]. Leukopenia characterized by neutropenia is usually a consistent finding in blood parasite infections such as trypanosomosis [41]. This finding also suggested that these cattle maybe repeatedly infected with these blood pathogens as shown in the farm records. GM-CSF behave in a paracrine manner by recruiting mature circulating neutrophils, monocytes and lymphocytes to enhance their functions in host defense (reviewed in [14]). Since blood pathogens are associated primarily with anaemia (haemolytic) or have more effect on the erythrocytic parameters, we suggest that the increase in serum GM-CSF levels (pro-inflammatory response) and proinflammatory immune response of GM-CSF characterized by high GM-CSF:IL10 ratio in the aforementioned cattle groups was in response to the degenerative left shift (higher immature neutrophils to mature neutrophils) reported in our previous study [4]. Though we do not have an evidence of secondary bacterial infection, serum GM-CSF, as a white blood cell growth factor is known to increase in animals with infections or inflammatory conditions associated with rapid exhaustion of mature neutrophils and increase demand of neutrophils or monocytes in the peripheral circulation. This demand, however, causes a stimulation of the bone marrow to release immature neutrophils, resulting in either a degenerative or regenerative left shift. Furthermore, GM-CSF stimulates the increase in monocyte and macrophage numbers whose primary function is to phagocytose invading pathogens and resolve the inflammation afterwards.

An anti-inflammatory cytokine immune response characterized by low IL-12:IL-10, IL-12:IL-4, IFN-γ:IL-10 and IFN-γ:IL-4 ratios predominated in the *C. Mycoplasma haemobos* and *T. orientalis* asymptomatic cattle. The anti-inflammatory cytokine response might have encouraged blood parasite persistence in the host due to inhibition of proinflammatory cytokines production by IL-10 and IL-4 activities as well as the cytotoxic effects of monocytes and macrophages [12].

Free oxygen radicals cause lipid peroxidation whose end product is malondialdehyde. The moderate increase in the serum malondialdehyde level in all the blood parasite infected cattle groups suggest a high degree of oxidative stress response during the course of the infection. This agrees with El-Ashker et al. [24] in *A. marginale* infection, Shiono et al. [42] in *T. sergenti* infection in cattle, Mishra et al. [43] and Parashar et al. [44] in *T. evansi* infection in Holstein cattle and horses, respectively. The measurement of glutathione peroxidase and superoxide dismutase activities are indirect ways to evaluate the status of antioxidant defense of the body [45].The increase in the serum activities of glutathione peroxidase and superoxide dismutase (antioxidant enzymes) reported in this study is in contrast with Esmaeilnejad et al. [46] who reported decreases in GPx and SOD activities in crossbred Holstein cattle naturally infected with *A. marginale,* and in [47] Rezaei and Dalir-Naghadeh [47] and Hassanpour et al. [48] who reported decreased GPx and SOD activities in Holstein cattle with tropical theileriosis. The increase in serum SOD activity observed in *A. platys* infection from this study was different from that of Himalini et al. [49] who reported a decrease in serum SOD activity in dogs with mixed *Babesia canis* and *Anaplasma platys/phagocytophilum* infection. These contrasting findings could be attributed to the time of sampling and stage of infection. The increase in serum GPx and SOD recorded in this study was because these enzymes are the first line of defense and also responded to increase in MDA concentration. Their roles are to mop up free radicals and reactive oxygen radicals that accumulate during a disease process, and thereby maintain the oxidant-antioxidant balance. Furthermore, there is a possibility that the animals are in close contact with arthropod vectors in the farm, thus the host immune system, nutrition and treatment might have played roles in impeding the effect of the blood parasites and hence increase the antioxidant status in the host and eliminating the reactive oxygen species thereafter [50]. Compatible with our findings, Grewal et al. [51] reported an increase in GPx activity in cattle with natural tropical theileriosis, but with no significant change in serum SOD. The increase in GPx and SOD could be attributed to the fact that these enzymes constitute a first line antioxidant defense system which plays key roles in the total defense mechanisms in biological systems such as intracellular destruction of lipid peroxides [52].

## 5. Conclusions

Pro-inflammatory cytokine immune response characterized by high serum GM-CSF:IL-10 ratio was the predominant finding in all the symptomatic natural blood parasite infected cattle except for *A. marginale.* Anti-inflammatory cytokine immune responses were notable findings in asymptomatic cattle infected with *C. M. haemobos* and *T. orientalis* characterized by low serum IL-12:IL-10, IL-12:IL-4, IFN-γ:IL-10 and IFN-γ:IL-4 ratios. Also, our findings clearly suggested that natural blood parasite infections can induce marked oxidative stress responses. The increased serum levels of malondialdehyde induced an increase in antioxidative stress responses characterized by increase in serum activities of SOD and GPx. A negative correlation existed between clinical symptoms and cytokine level in all the infected cattle groups. Furthermore, a negative correlation existed between gender and IL-10 in the *T. orientalis*-infected cattle, and between age and IL-4 in the *T. sinensis* infected cattle. Also, the highest serum SOD response was found in the *C. M. haemobos*, *A. marginale* and *A. platys* infected cattle sampled from private farm. Therefore, high serum GM-CSF:IL-10 in symptomatic cattle, low serum IL-12:IL-10, IL-12:IL-4, IFN-γ:IL-10 and IFN-γ:IL-4 ratios in asymptomatic cattle, and high serum MDA level alongside increased SOD and GPx activities in both symptomatic and asymptomatic cattle could be useful predictive tools for outcome of natural blood parasite infections in cattle.

## Figures and Tables

**Table 1:** Forward and reverse primer sequences and thermal cycling conditions for the PCR detection of cattle blood parasites. Thermal cycling conditions include initial denaturing (ID), denaturing (D), annealing (A), extension (E), and final extension (FE).

Blood Parasites	Gene of Interest	Primer Sequences (5′–3′) Forward (F) and Reverse (R)	Thermocycling Conditions	Base Pair (bp)	References
*T. sinensis*	MPSP	F-CACTGCTATGTTGTCCAAGAGATATT R- AATGCGCCTAAAGATAGTAGAAAAC	ID: 94 °C/3 min D: 94 °C/1 min A: 56 °C/1 min E: 72 °C/1 min No. of cycles:40 FE: 72 °C/7 min	887	[28]
*A. marginale*	MSP4	F- CATCTCCCATGAGTCACGAAGTGGC R- GCTGAACAGGAATCTTGCTCCAAG	ID: 95 °C/5 min D: 95 °C/1 min A: 65 °C/2 min E: 72 °C/1 min No. of cycles:40 FE: 72 °C/10 min	761	[29]
*T. orientalis*	MPSP	F- CTTTGCCTAGGATACTTCCT R -ACGGCAAGTGGTGAGAACT	ID: 94 °C/4 min D: 94 °C/1 min A: 63 °C/1 min E: 72 °C/1 min No. of cycles:40 FE: 72 °C/7 min	776	[30]
*T. evansi*	RoTaT 1.2 VSG	F- 5′-GCGGGGTGTTTAAAGCAATA-3′ R- 5′-ATTAGTGCTGCGTGTGTTCG-3′	ID: 94 °C/4 min D: 94 °C/1 min A: 59 °C/1 min E: 72 °C/1 min No. of cycles:40 FE: 72 °C/5 min	208	[31]
*Candidatus Mycoplasma haemobos*	16 SrRNA	F- GAGTTAGTTATTAAAGCTTTAT R- ATTCATGAGGTACTATCAGTTG	ID: 94 °C/5 min D: 94 °C/30 s A: 55 °C/30 s E: 72 °C/30 s No. of cycles:40 FE: 72 °C/7 min	279	[32]
*A. platys*	16SrRNA	PLATYS F- AAGTCGAACGGATTTTTGTC PLATYS R -CTTTAACTTACCGAACC	ID: 95 °C/5 min D: 95 °C/30 s A: 55 °C/30 s E: 72 °C/90 s No. of cycles:40 FE: 72 °C/5 min	504	[33]

**Table 2:** Detection rate of blood parasites in the sampled cattle.

Blood Parasites	No. of Cattle	Occurrence (%) (95% Confidence Interval)
*Candidatus Mycoplasma haemobos*	70	53.9 (45.3–62.2)
*Theileria orientalis*	65	50 (41.5–58.5)
*Anaplasma marginale*	49	37.8 (29.8–46.3)
*Theileria sinensis*	32	24.6 (18.0–32.3)
*Trypanosoma evansi*	4	3.08 (1.20–7.64)
*Anaplasma platys*	7	5.38 (2.63–10.7)

Number of sampled cattle = 130; Number of clinically healthy cattle = 20.

**Table 3:** The serum inflammatory cytokine and oxidant/antioxidant levels in symptomatic and asymptomatic cattle naturally infected with *Candidatus Mycoplasma haemobos*.

Proinflammatory/ Anti-Inflammatory Cytokines	*C. Mycoplasma haemobos* Infected Cattle (Symptomatic) (*n* = 3)	*C. Mycoplasma haemobos* Infected Cattle (Asymptomatic) (*n* = 12)	Clinically Healthy Cattle (*n* = 8)
Interleukin 12 (ng/L)	4.11 ± 0.05 ^b^	5.71 ± 1.19 ^c^	2.36 ± 0.08 ^a^
Interleukin 10 (ng/L)	40.78 ± 3.87 ^b^	50.45 ± 3.27 ^b^	19.53 ± 0.43 ^a^
GM-CSF (pg/mL)	745.73 ± 3.02 ^b^	717.75 ± 2.81 ^b^	81.68 ± 1.26 ^a^
Interleukin 4 (ng/L)	864.70 ± 13.36 ^c^	795.64 ± 12.74 ^b^	349.85 ± 3.05 ^a^
Interferon-γ (pg/mL)	163.18 ± 1.05 ^a^	184.50 ± 5.26 ^b^	167.42 ± 4.38 ^a^
**Oxidant/antioxidant Markers**			
Malondialdehyde (nM/mL)	3.70 ± 1.60 ^c^	2.51 ± 0.16 ^b^	1.37 ± 0.02 ^a^
Superoxide dismutase (U/mL)	945.36 ± 4.87 ^b^	977.16 ± 9.72 ^b^	546.6 ± 4.64 ^a^
Glutathione peroxidase (U/mL)	138.24 ± 2.10 ^b^	138.63 ± 1.59 ^b^	54.40 ± 0.45 ^a^

^a,b,c^ Different superscripts in a row refer to significant differences in the parameters between infected cattle groups and clinically healthy cattle (*p* < 0.05).

**Table 4:** The serum inflammatory cytokine and oxidant/antioxidant levels in symptomatic and asymptomatic cattle in cattle naturally infected by *Theileria orientalis*.

Proinflammatory/ Anti-Inflammatory Cytokines	*T. orientalis* Infected Cattle (Symptomatic) (*n* = 4)	*T. orientalis* Infected Cattle (Asymptomatic) (*n* = 9)	Clinically Healthy Cattle (*n* = 8)
Interleukin 12 (ng/L)	5.83 ± 0.53 ^b^	5.20 ± 0.24 ^b^	2.36 ± 0.08 ^a^
Interleukin 10 (ng/L)	45.87 ± 5.16 ^b^	38.60 ± 2.41 ^b^	19.53 ± 0.43 ^a^
GM-CSF (pg/mL)	454.55 ± 8.74 ^b^	473.88 ± 8.97 ^b^	81.68 ± 1.26 ^a^
Interleukin 4 (ng/L)	689.50 ± 10.34 ^b^	705.15 ± 11.64 ^b^	349.85 ± 3.05 ^a^
Interferon-γ (pg/mL)	276.97 ± 16.35 ^b^	261.15 ± 10.87 ^b^	167.42 ± 4.38 ^a^
**Oxidant/antioxidant markers**			
Malondialdehyde (nM/mL)	3.97 ± 0.93 ^b^	3.01 ± 0.45 ^b^	1.37 ± 0.02 ^a^
Superoxide dismutase (U/mL)	854.31 ± 20.83 ^b^	921.80 ± 21.77 ^b^	546.6 ± 4.64 ^a^
Glutathione peroxidase (U/mL)	188.83 ± 6.64 ^b^	186.00 ± 3.97 ^b^	54.40 ± 0.45 ^a^

^a,b^ Different superscripts in a row refers to significant differences in the parameters between infected cattle groups and clinically healthy cattle (*p* < 0.05).

**Table 5:** The serum inflammatory cytokine and oxidant/antioxidant levels in symptomatic and asymptomatic cattle naturally infected by *Anaplasma marginale*.

Proinflammatory/ Anti-Inflammatory Cytokines	*Anaplasma marginale* Infected Cattle (Symptomatic) (*n* = 3)	*Anaplasma marginale* Infected Cattle (Asymptomatic) (*n* = 3)	Clinically Healthy Cattle (*n* = 8)
Interleukin 12 (ng/L)	20.03 ± 1.80 ^b^	37.35 ± 1.12 ^c^	2.36 ± 0.08 ^a^
Interleukin 10 (ng/L)	39.50 ± 3.18 ^a^	342.53 ± 12.41 ^b^	19.53 ± 0.43 ^a^
GM-CSF (pg/mL)	515.62 ± 12.41 ^b^	517.80 ± 19.76 ^b^	81.68 ± 1.26 ^a^
Interleukin 4 (ng/L)	749.66 ± 9.66 ^b^	964.86 ± 4.35 ^b^	349.85 ± 3.05 ^a^
Interferon-γ (pg/mL)	291.96 ± 29.48 ^b^	373.91 ± 28.045 ^c^	167.42 ± 4.38 ^a^
**Oxidant/Antioxidant Markers**			
Malondialdehyde (nM/mL)	3.88 ± 1.81 ^b^	2.18 ± 0.05 ^a^	1.37 ± 0.02 ^a^
Superoxide dismutase (U/mL)	1236.33 ± 10.88 ^b^	932.99 ± 17.24 ^b^	546.6 ± 4.64 ^a^
Glutathione peroxidase (U/mL)	145.20 ± 1.12 ^b^	277.74 ± 9.49 ^c^	54.40 ± 0.45 ^a^

^a,b,c^ Different superscripts in a row refers to significant differences in the parameters between infected cattle groups and clinically healthy cattle (*p* < 0.05).

**Table 6:** The serum inflammatory cytokines and oxidant/antioxidant levels in symptomatic and asymptomatic cattle naturally infected with *Theileria sinensis*.

Proinflammatory/ Anti-Inflammatory Cytokines	*Theileria sinensis* Infected Cattle (Symptomatic) (*n* = 3)	*Theileria sinensis* Infected Cattle (Asymptomatic) (*n* = 3)	Clinically Healthy Cattle (*n* = 8)
Interleukin 12 (ng/L)	17.88 ± 3.35 ^b^	3.50 ± 0.66 ^a^	2.36 ± 0.08 ^a^
Interleukin 10 (ng/L)	52.36 ± 1.60 ^c^	40.92 ± 1.71 ^b^	19.53 ± 0.43 ^a^
GM-CSF (pg/mL)	340.63 ± 15.24 ^b^	318.09 ± 12.77 ^b^	81.68 ± 1.26 ^a^
Interleukin 4 (ng/L)	965.24 ± 17.29 ^c^	686.64 ± 6.41 ^b^	349.85 ± 3.05 ^a^
Interferon-γ (pg/mL)	352.90 ± 14.50 ^b^	256.05 ± 12.94 ^ab^	167.42 ± 4.38 ^a^
**Oxidant/antioxidant markers**			
Malondialdehyde (nM/mL)	5.10 ± 0.05 ^b^	4.00 ± 1.51 ^b^	1.37 ± 0.02 ^a^
Superoxide dismutase (U/mL)	969.90 ± 5.69 ^c^	937.66 ± 13.03 ^b^	546.6 ± 4.64 ^a^
Glutathione peroxidase (U/mL)	242.53 ± 12.82 ^b^	235.91 ± 6.34 ^b^	54.40 ± 0.45 ^a^

^a,b,c^ Different superscripts in a row refers to significant differences in the parameters between infected cattle groups and clinically healthy cattle (*p* < 0.05).

**Table 7:** The serum inflammatory cytokines and oxidant/antioxidant levels in symptomatic and asymptomatic cattle naturally infected with *Trypanosoma evansi*.

Proinflammatory/ Anti-Inflammatory Cytokines	*T. evansi* Infected Cattle (Symptomatic) (*n* = 4)	Clinically Healthy Cattle (*n* = 8)
Interleukin 12 (ng/L) *	8.96 ± 0.53	2.36 ± 0.08
Interleukin 10 (ng/L) *	71.00 ± 1.83	19.53 ± 0.43
GM-CSF (pg/mL) *	555.42 ± 20.7	81.68 ± 1.26
Interleukin 4 (ng/L) *	770.41 ± 14.82	349.85 ± 3.05
Interferon-γ (pg/mL) *	277.02 ± 5.41	167.42 ± 4.38
Oxidant/Antioxidant Markers		
Malondialdehyde (nM/mL) *	13.73 ± 0.15	1.37 ± 0.02
Superoxide dismutase (U/mL) *	1162.76 ± 19.01	546.6 ± 4.64
Glutathione peroxidase (U/mL) *	128.51 ± 2.10	54.40 ± 0.45

* Asterisk superscript on a parameter indicates significant difference *p* < 0.05.

**Table 8:** The serum inflammatory cytokine and oxidant/antioxidant levels in symptomatic and asymptomatic cattle in cattle naturally infected by *Anaplasma platys*.

Proinflammatory/ Anti-Inflammatory Cytokines	*Anaplasma platys* Infected Cattle (Symptomatic) (*n* = 3)	Clinically Healthy Cattle (*n* = 8)
Interleukin 12 (ng/L) *	58.60 ± 0.07	2.36 ± 0.08
Interleukin 10 (ng/L) *	665.04 ± 5.05	19.53 ± 0.43
GM-CSF (pg/mL) *	422.28 ± 10.93	81.68 ± 1.26
Interleukin 4 (ng/L) *	1279.33 ± 20.12	349.85 ± 3.05
Interferon-γ (pg/mL) *	350.12 ± 4.26	167.42 ± 4.38
Oxidant/Antioxidant Markers		
Malondialdehyde (nM/mL) *	5.84 ± 0.15	1.37 ± 0.02
Superoxide dismutase (U/mL) *	1561.46 ± 15.74	546.6 ± 4.64
Glutathione peroxidase (U/mL) *	343.14 ± 14.09	54.40 ± 0.45

* Asterisk superscript on a parameter indicates significant difference *p* < 0.05.

## Data Availability

The data presented in this study are available on request from the corresponding author.

## Supplementary Materials

The following are available online at https://www.mdpi.com/article/10.3390/ani11082235/s1, Table S1. Correlation between clinical symptoms, age and sex, and cytokines in cattle naturally infected with *Candidatus Mycoplasma haemobos.* Table S2. Correlation between clinical symptoms, age and sex, and cytokines in cattle naturally infected with *Theileria orientalis.* Table S3. Correlation between clinical symptoms, sex, and cytokines levels in cattle naturally infected with *Anaplasma marginale.* Table S4. Correlation between clinical symptoms, age, sex, and cytokine levels in cattle naturally infected with *Theileria sinensis.* Table S5. Correlation between clinical symptoms and cytokine levels in cattle naturally infected with *Trypanosoma evansi.* Table S6. Correlation between clinical symptoms, sex, and cytokine levels in cattle naturally infected with *Anaplasma platys.* Table S7. Effect of farm ownership and age on oxidative and antioxidative stress responses in cattle naturally infected with *Candidatus Mycoplasma haemobos.* Table S8. Effect of farm ownership on oxidative and antioxidative stress responses in cattle naturally infected with *Theileria orientalis.* Table S9. Effect of farm ownership on oxidative and antioxidative stress responses in cattle naturally infected with *A. marginale.* Table S10. Effect of farm ownership and age on oxidative and antioxidative stress responses in cattle naturally infected with *Theileria sinensis.* Table S11. Effect of farm ownership on oxidative and antioxidative stress responses in cattle naturally infected with *A. platys.*
