# Supplementary material for: High Granulocyte-Macrophage Colony Stimulating Factor to Interleukin 10 Ratio and Marked Antioxidant Enzyme Activities Predominate in Symptomatic Cattle Naturally Infected with Candidatus Mycoplasma haemobos, Theileria orientalis, Theileria sinensis and Trypanosoma evansi
Source: Animals (Basel). 2021 Jul 29;11(8):2235. doi: 10.3390/ani11082235 (PMC8388426; doi:10.3390/ani11082235)
Supplement: Supplementary file 1 [file animals-11-02235-s001.zip › animals-1171777-supplementary.pdf]

animals

MDPI

Table S1. Correlation between clinical symptoms, age and sex, and cytokines in cattle naturally infected with *Candidatus Mycoplasma haemobos.*

Proinflammatory/ Anti-inflammatory Cytokines	Age		Sex		Clinical Symptoms	
	Correlation	*p* Value	Correlation	*p* Value	Correlation	*p* Value
Interleukin 12 (ng/L)	−0.231	0.127	−0.007	0.874	**−0.685**	**0.001**
Interleukin 10 (ng/L)	−0.652	0.543	−0.310	0.632	**−0.618**	**0.003**
GM-CSF (pg/mL)	0.006	0.964	0.010	0.936	**−0.315**	**0.006**
Interleukin 4 (ng/L)	−0.143	0.247	0.089	0.476	**0.409**	**0.000**
Interferon-γ (pg/mL)	0.163	0.187	0.054	0.663	−0.088	0.451

Numbers with boldface indicates association ($p < 0.05$) between factors (age, sex and clinical symptoms) and cytokine level in the blood parasite infected cattle.

Table S2. Correlation between clinical symptoms, sex, and cytokines in cattle naturally infected with *Theileria orientalis.*

Proinflammatory/ Anti-inflammatory Cytokines	Sex		Clinical Symptoms	
	Correlation	*p* value	Correlation	*p* Value
Interleukin 12 (ng/L)	−0.006	0.963	**−0.323**	**0.007**
Interleukin 10 (ng/L)	−0.294	0.022	**−0.374**	**0.002**
GM-CSF (pg/mL)	0.163	0.209	**−0.279**	**0.020**
Interleukin 4 (ng/L)	0.072	0.581	**−0.406**	**0.001**
Interferon-γ (pg/mL)	0.053	0.686	**−0.337**	**0.005**

Numbers with boldface indicates association ($p < 0.05$) between factors (sex and clinical symptoms) and cytokine level in the blood parasite infected cattle.

Table S3. Correlation between clinical symptoms, sex, and cytokines levels in cattle naturally infected with *Anaplasma marginale.*

Proinflammatory/ Anti-inflammatory Cytokines	Sex		Clinical symptoms	
	Correlation	*p* value	Correlation	*p* Value
Interleukin 12 (ng/L)	−0.006	0.963	**−0.323**	**0.007**
Interleukin 10 (ng/L)	−0.294	0.022	**−0.374**	**0.002**
GM-CSF (pg/mL)	0.163	0.209	**−0.279**	**0.020**
Interleukin 4 (ng/L)	0.072	0.581	**−0.406**	**0.001**
Interferon-γ (pg/mL)	0.053	0.686	**−0.337**	**0.005**

Numbers with boldface indicates association ($p < 0.05$) between factors (age and clinical symptoms) and cytokine level in the blood parasite infected cattle.

Table S4. Correlation between clinical symptoms, age, sex, and cytokine levels in cattle naturally infected with *Theileria sinensis.*

Proinflammatory/ Anti-inflammatory Cytokines	Age		Sex		Clinical Symptoms	
	Correlation	*p* value	Correlation	*p* value	Correlation	*p* value
Interleukin 12 (ng/L)	0.129	0.491	−0.075	0.686	**−0.444**	**0.005**
Interleukin 10 (ng/L)	0.271	0.141	−0.156	0.403	**−0.433**	**0.006**
GM-CSF (pg/mL)	0.082	0.661	0.177	0.342	**−0.600**	**0.000**
Interleukin 4 (ng/L)	**−0.512**	**0.003**	0.033	0.862	**−0.488**	**0.002**
Interferon-γ (pg/mL)	0.103	0.081	0.158	0.396	**−0.441**	**0.005**

Numbers with boldface indicates association ($p < 0.05$) between factors (age and clinical symptoms) and cytokine level in the blood parasite infected cattle.

Table S5. Correlation between clinical symptoms and cytokine levels in cattle naturally infected with *Trypanosoma evansi.*

Proinflammatory/ Anti-inflammatory Cytokines	Clinical Symptoms	
	Correlation	*p* Value
Interleukin 12 (ng/L)*	**−0.984**	**0.000**
Interleukin 10 (ng/L)*	**−0.996**	**0.000**
GM-CSF (pg/mL)*	**−0.996**	**0.000**
Interleukin 4 (ng/L)*	**0.997**	**0.000**
Interferon-γ (pg/mL)*	**−0.978**	**0.000**

*Asterisk superscript on a parameter indicates significant difference at $p < 0.05$. Numbers with boldface indicates association ($p < 0.05$) between clinical symptoms and cytokine level in the blood parasite infected cattle.

Table S6. Correlation between clinical symptoms, sex, and cytokine levels in cattle naturally infected with *Anaplasma platys.*

Proinflammatory/ Anti-inflammatory Cytokines	Sex		Clinical Symptoms	
	Correlation	*p* value	Correlation	*p* value
Interleukin 12 (ng/L)	0.403	0.072	**−0.785**	**0.002**
Interleukin 10 (ng/L)	0.371	0.410	**−0.670**	**0.005**
GM-CSF (pg/mL)	−0.367	0.418	**−0.941**	**0.000**
Interleukin 4 (ng/L)	0.243	0.600	**−0.929**	**0.000**
Interferon-γ (pg/mL)	0.501	0.252	**−0.812**	**0.396**

Numbers with boldface indicates association ($p < 0.05$) between factors (sex and clinical symptoms) and cytokine level in the blood parasite infected cattle.

Table S7. Effect of farm ownership and age on oxidative and antioxidative stress responses in cattle naturally infected with *Candidatus Mycoplasma haemobos.*

Parameters	Private Farm	Government Farm	*p* Value
MDA (nM/ml)	2.58 ± 0.19	2.39 ± 0.08	0.344
SOD (U/ml) *	941.85 ± 15.51	876.49 ± 21.35	0.017
GPx (U/ml)	178.12 ± 11.2	206.20 ± 13.56	0.118
	**Young**	**Adult**	***p* Value**
MDA (nM/ml)	2.29 ± 0.20	2.53 ± 0.10	0.541
SOD (U/ml)	958.99 ± 13.32	936.03 ± 15.36	0.710
GPx (U/ml)	191.81 ± 12.93	192.01 ± 7.73	0.995

*Asterisk superscript on a parameter indicates significant difference, $p < 0.05$.

Table S8. Effect of farm ownership on oxidative and antioxidative stress responses in cattle naturally infected with *Theileria orientalis.*

Parameters	Private Farm	Government Farm	*p* value
MDA (nM/ml)	2.89 ± 0.31	2.59 ± 0.14	0.313
SOD (U/ml)	937.93 ± 21.44	937.71 ± 25.57	0.996
GPx (U/ml)	200.08 ± 10.7	191.54 ±± 8.11	0.546

Oxidative stress response was not computed for age because all *T. orientalis* infected cattle were adults.

Table S9. Effect of farm ownership on oxidative and antioxidative stress responses in cattle naturally infected with *A. marginale.*

Parameters	Private farm	Government farm	*p* Value
MDA (nM/ml)	2.47 ± 0.28	2.40 ± 0.08	0.773
SOD (U/ml)	989.61 ± 12.90	844.98 ± 16.28	0.003
GPx (U/ml)	216.63 ± 19.63	194.90 ± 13.52	0.353

Oxidative stress response was not computed for age because all *A. marginale* infected cattle were adults.

Table S10. Effect of farm ownership and age on oxidative and antioxidative stress responses in cattle naturally infected with *Theileria sinensis.*

Parameters	Private Farm	Government Farm	*p* Value
MDA (nM/ml)	3.49 ± 0.58	2.67 ± 0.95	0.090
SOD (U/ml)	929.38 ± 20.91	877.85 ± 30.53	0.383
GPx (U/ml)	216.90 ± 18.82	195.18 ± 12.76	0.407
**Age**	**Young**	**Adult**	***p* Value**
MDA (nM/ml)	2.38 ± 0.36	2.89 ± 0.22	0.549
SOD (U/ml)	938.44 ± 6.85	886.11 ± 25.83	0.605
GPx (U/ml)	248.62 ± 5.59	196.74 ± 11.22	0.242

Table S11. Effect of farm ownership on oxidative and antioxidative stress responses in cattle naturally infected with *A. platys.*

Parameters	Private Farm	Government Farm	*p* value
MDA (nM/ml)	5.84 ± 0.15	4.59 ± 0.83	0.262
SOD (U/ml) *	1561.46 ± 15.74	1029.26 ± 13.14	0.033
GPx (U/ml)	343.14 ± 14.09	271.51 ± 12.63	0.058

Oxidative stress response was not computed for age because all *A. platys* infected cattle were adults.
